# The AHR repressor limits expression of antimicrobial genes but not AHR-dependent genes in intestinal eosinophils

**DOI:** 10.1093/jleuko/qiae105

**Published:** 2024-05-03

**Authors:** Heike Weighardt, Michael Shapiro, Michelle Mayer, Irmgard Förster, Brigitta Stockinger, Nicola Laura Diny

**Affiliations:** Immunology and Environment, Life and Medical Sciences Institute, University of Bonn, Carl-Troll-Straße 31, 53115 Bonn, Germany; AhR Immunity Lab, The Francis Crick Institute, 1 Midland Road, London NW1 1AT, United Kingdom; Immunology and Environment, Life and Medical Sciences Institute, University of Bonn, Carl-Troll-Straße 31, 53115 Bonn, Germany; Immunology and Environment, Life and Medical Sciences Institute, University of Bonn, Carl-Troll-Straße 31, 53115 Bonn, Germany; AhR Immunity Lab, The Francis Crick Institute, 1 Midland Road, London NW1 1AT, United Kingdom; AhR Immunity Lab, The Francis Crick Institute, 1 Midland Road, London NW1 1AT, United Kingdom; Institute of Clinical Chemistry and Clinical Pharmacology, University Hospital Bonn, Venusberg-Campus 1, 53127 Bonn, Germany

**Keywords:** aryl hydrocarbon receptor, eosinophils, intestine, mucosal immunology, transcription factor

## Abstract

Intestinal eosinophils express the aryl hydrocarbon receptor (AHR), an environmental sensor and ligand-activated transcription factor that responds to dietary or environmental ligands. AHR regulates tissue adaptation, survival, adhesion, and immune functions in intestinal eosinophils. The AHR repressor (AHRR) is itself induced by AHR and believed to limit AHR activity in a negative feedback loop. We analyzed gene expression in intestinal eosinophils from wild-type and AHRR knockout mice and found that AHRR did not suppress most AHR-dependent genes. Instead, AHRR limited the expression of a distinct small set of genes involved in the innate immune response. These included S100 proteins, antimicrobial proteins, and alpha-defensins. Using bone marrow–derived eosinophils, we found that AHRR knockout eosinophils released more reactive oxygen species upon stimulation. This work shows that the paradigm of AHRR as a repressor of AHR transcriptional activity does not apply to intestinal eosinophils. Rather, AHRR limits the expression of innate immune response and antimicrobial genes, possibly to maintain an anti-inflammatory phenotype in eosinophils when exposed to microbial signals in the intestinal environment.

## Teaser

The aryl hydrocarbon receptor repressor acts as a transcriptional repressor of a distinct set of antimicrobial genes and limits reactive oxygen species production in eosinophils, possibly maintaining their intestinal anti-inflammatory phenotype.

## 1. Introduction

Eosinophils are increasingly recognized as important immune cells in the intestinal mucosa. They contribute to epithelial barrier function and antimicrobial defense against pathogens, modulate other immune cells, and remodel the extracellular matrix.^1–10^ To perform these functions, eosinophils adopt an intestine-specific gene expression program.^7,9,10^ We could previously show that the aryl hydrocarbon receptor (AHR) mediates part of this tissue-specific gene expression program and controls several important aspects of intestinal eosinophil biology.^7^ AHR regulates eosinophil adhesion to and degradation of extracellular matrix, degranulation, lifespan, and their ability to maintain regulatory T cells. The importance of AHR and AHR-regulated genes in intestinal eosinophils has since been confirmed by multiple studies.^8–10^

AHR has long been recognized as a critical transcription factor in the regulation of intestinal homeostasis. It is expressed in many immune and stromal cells in the intestinal mucosa and plays a key role in maintaining the epithelial barrier, antibacterial defense through T helper 17/ILC3-epithelial crosstalk and limiting the development of colorectal cancer by promoting epithelial differentiation.^7,11–17^ As a transcription factor, AHR can regulate the expression of hundreds of genes, often in a cell type–specific manner.^7,18–22^ As a result, AHR has diverse functions in physiological and pathologic settings in the intestine.

AHR activity is controlled by the availability of ligands, which may be derived from the diet or environmental toxins.^12^ Naturally occurring ligands include the high affinity ligand FICZ, which is generated by photolysis of L-tryptophan. Industrial processes generate a range of xenobiotic ligands, such as 3-MC or TCDD. AHR is retained in a chaperone complex in the cytoplasm and released upon ligand binding. It translocates to the nucleus, dimerizes with ARNT, and induces transcription of target genes by binding to dioxin-response elements. Among these target genes are several negative feedback regulators. Cytochrome P450 enzymes CYP1A1, CYP1A2, and CYP1B1 can biotransform natural AHR ligands for degradation, thereby limiting the activity of AHR.^23,24^ TIPARP mediates AHR nuclear export and subsequent proteasomal degradation.^25,26^ The AHR repressor (AHRR) forms another negative feedback mechanism. AHR and AHRR are basic helix-loop-helix proteins with a dioxin-response element–binding domain and both bind to ARNT. AHRR contains only 1 PAS (Per-Arnt-Sim) domain and lacks the PAS B domain that mediates ligand binding in AHR.^27^ In vitro experiments showed that overexpression of AHRR inhibits transactivation of AHR and established the dogma that AHRR competes with AHR for ARNT binding and thereby limits its ability to induce target gene expression.^27^ Interaction of AHRR with ARNT has been confirmed by crystal structure analysis,^28^ but overexpression of ARNT did not rescue the effects of AHRR overexpression on inhibition of AHR activity.^29^ AHRR can also directly bind AHR, and its DNA-binding domain is not necessary to repress AHR activity.^29^ Chromatin immunoprecipitation sequencing analysis of a TCDD-treated breast cancer cell line revealed thousands of binding sites for both AHR and AHRR, but only about 70% of these sites overlapped, and nearly 1,000 binding sites unique to AHRR were identified.^19^ In primary human fibroblasts and AHRR knockout (AHRR-KO) mouse embryonic fibroblasts, repression of CYP1A1 enzymatic activity was independent of AHRR expression.^30^ These data suggest that AHRR molecular functions may not be limited to suppressing AHR activation.

More recent in vivo work has shown that AHRR is expressed in several intestinal immune cells such as macrophages, dendritic cells, intraepithelial lymphocytes, and γδ T cells but not in epithelial cells.^16,31^ AHRR is induced by dietary ligands in intestinal immune cells and AHR ligands protect from dextran sulfate sodium colitis, while both AHR-KO and AHRR-KO mice experience increased disease severity.^31^ A recent study showed that lack of AHRR in intraepithelial lymphocytes resulted in increased oxidative stress due to CYP1A1 overactivity in line with the paradigm of AHRR-mediated repression of AHR target genes.^32^ This implies that AHR activity needs to be tightly controlled and that both lack of AHR activity and AHR overactivation in AHRR-deficient mice or lack of AHRR-dependent effects have negative consequences for intestinal immune cells and tissue homeostasis. We previously demonstrated that AHRR is highly expressed in intestinal but not bone marrow eosinophils, and its expression is dependent on AHR.^7^ However, the role of AHRR in intestinal eosinophils and whether it regulates the activity of AHR is unknown.

## 2. Methods

### 2.1 Mice

*Ahrr* ^EGFP/EGFP^ mice and the wild-type (WT) control animals in the corresponding experiments were bred and maintained at the animal facility at the LIMES (Life and Medical Sciences) Institute at the University of Bonn. Male and female mice aged between 6 and 16 wk were used for the experiments and were bred according to German guidelines for animal care. All experiments were performed according to German and institutional guidelines for animal experimentation.

All other mice were bred and maintained at the Francis Crick Institute. *Myd88*^−/−^*Trif*^−/−^ mice (Myd88^tm1Aki^, Ticam1^tm1Aki33,34^) were bred and maintained in isolators. WT, *Ahr*^−/−^,^35^*Ahrr*^−/−^ (*Ahrr^tm1b(KOMP)Wtsi^)*, and *Cyp1a1*^Cre/+^*Rosa26*^YFP13^ were bred and maintained in individually ventilated cages under specific pathogen-free conditions. All mice were bred and maintained according to the protocols approved by the UK Home Office and the ethics committee (AWERP) of the Francis Crick Institute. Both male and female mice between 6 and 14 wk of age were used in experiments. All mice were on the C57BL/6 background. Within experiments, mice were sex-matched and age-matched within 3 wk. All mouse strains were first rederived via embryo or sperm transfer into the Francis Crick Institute breeding facility, and none were directly purchased from external vendors. Mice of different genotypes were not littermates and were not cohoused.

### 2.2 3-MC Administration

3-MC was administered by intraperitoneal injection at 26.5 mg/kg bodyweight in corn oil. Mice were analyzed 3 d after injection. Control mice were injected with corn oil only.

### 2.3 Bone marrow–derived eosinophils

Bone marrow–derived eosinophils (BMDEos) were cultured as previously described.^36^ In brief, bone marrow was isolated under a laminar flow hood, and cells were seeded at 2 × 10^6^ cells/mL in BMDEo medium: RPMI1640 (Corning; #10-040-CV) supplemented with 20% fetal calf serum (FCS), penicillin/streptomycin, 2 mM L-Glutamine, 25 mM HEPES, nonessential amino acids, 1 mM sodium pyruvate, and 55 nM 2-mercaptoethanol. For the first 4 d 100 ng/mL stem cell factor and 100 ng/mL FLT3L (both PeproTech) were added to the medium. Media was changed on days 4, 8, and 11 to fresh medium with 10 ng/mL interleukin (IL)-5 (R&D Systems) and cell concentrations were readjusted on days 8 and 11 to 1 × 10^6^ cells/mL. On day 8, cells were transferred to a new flask. Cells were cultured at 37 °C, 5% CO_2_.

### 2.4 EROD assay

On day 14 of culture BMDEos were seeded at 1 × 10^6^ cells per 300 µL in 96 well plates and cultured with different concentrations of FICZ for 4 h. Cells were washed in phosphate-buffered saline (PBS) and resuspended in 100 µL phosphate buffer (50 mM NaHPO_4_, 50 mM NaH_2_PO_4_, pH 8.0) with 2 µM resorufin ethyl ether (Sigma-Aldrich; #E3763). Cells were incubated for 30 min. In this time resorufin ethyl ether is converted to resorufin by the CYP1A1 and CYP1B1 enzymes. As well, 75 µL acetonitrile containing fluorescamine (150 µg/mL) was added to stop the reaction and resorufin fluorescence was measured 535 nm excitation, 590 nm emission. Fluorescamine fluorescence was measured at 390 nm excitation, 485 nm emission. Serial dilutions of resorufin (Sigma-Aldrich; #424455) and bovine serum albumin were measured in parallel to generate standard curves. Resorufin concentration was normalized to protein content.

### 2.5 Generation of single-cell suspensions from different tissues

Cells from spleen were isolated by mashing through a 70 µm filter, followed by ACK lysis, washing in PBS, and filtration through a 70 µm filter. Intestinal lamina propria cells were isolated by first cleaning the intestines of fecal content. Intestines were cut longitudinally and washed in PBS, and the epithelial layer removed by incubating for 40 min at 37 °C, 200 rpm in Hank’s Balanced Salt Solution, 5% FCS, 2 mM EDTA. Intestines were washed in PBS, minced, and digested for 25 to 30 min at 37 °C, 200 rpm in Hank’s Balanced Salt Solution, 5% FCS, 1.5 mg/mL Collagenase VIII, and 80 µg/mL DNase I. Cells were filtered through a 100 µm filter, washed in fluorescence-activated cell sorting (FACS) buffer, filtered through a 70 µm filter, and washed again. Unless otherwise noted, cells were centrifuged at 300 *g* for 5 min at 4 °C.

### 2.6 Flow cytometry

Single-cell suspensions were prepared as described previously and incubated with anti-CD16/32 (eBioscience) and fixable Live/Dead cell stain (Thermo Fisher Scientific) for 30 min at 4 °C and washed in PBS. Cells were then incubated in FACS buffer with directly conjugated antibodies for 30 min at 4 °C. Cells were washed in FACS buffer and optionally fixed in 3% paraformaldehyde in PBS for 30 min to 18 h at 4 °C. Samples were acquired on a BD Fortessa instrument (BD Biosciences) and analyzed using FlowJo v10 (TreeStar). Samples were gated on single cells using forward scatter area/forward scatter height and side scatter area/side scatter height and to exclude debris on forward scatter area/side scatter area. Dead cells were excluded before gating based on antibody staining to identify cell populations as described in the respective figures. Intestinal eosinophils were generally identified as CD45^+^CD11b^+^MHC-II^−^Siglec-F^+^SSC^hi^ cells.

### 2.7 Cell sorting

**Fig. 1.**
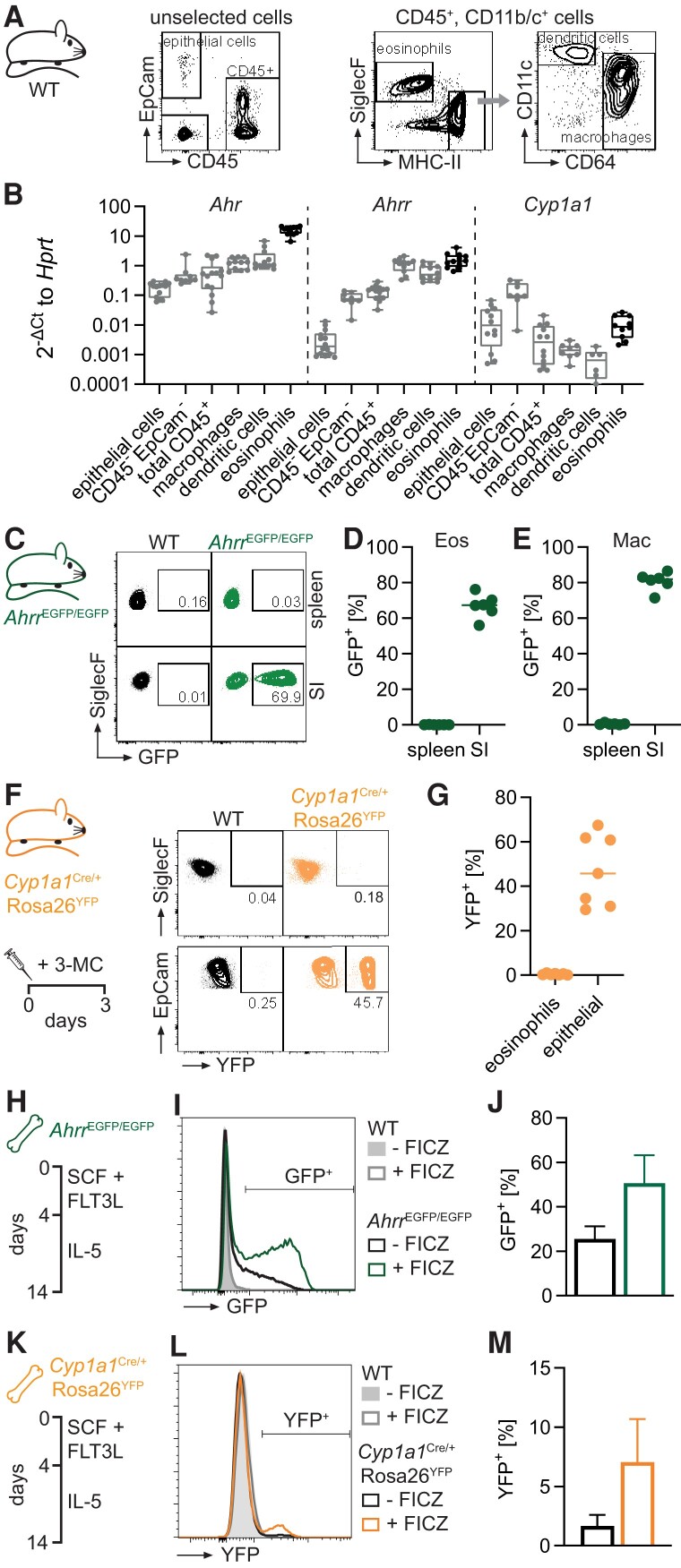
AHRR is highly expressed in intestinal eosinophils. (A) Representative flow cytometry plots of sorted cell populations from the small intestine. Unselected cells were used to sort epithelial cells, CD45^+^ cells, and CD45^−^EpCam^−^ cells. Myeloid cells were first enriched by positive magnetic-activated cell sorting selection with anti-CD11b and anti-CD11c microbeads. All populations were pregated on live, single cells. (B) Gene expression was determined by qPCR in sorted cells and normalized to *Hprt*. Data are pooled from 3 independent experiments for n = 8 to 14 biological replicates. Data from one experiment were previously reported in Diny et al.^7^ (C) Representative flow cytometry plots of splenic and small intestinal eosinophils from WT and *Ahrr*^EGFP/EGFP^ mice. (D, E) Frequency of AHRR-EGFP^+^ eosinophils and macrophages. Data are pooled from 2 independent experiments for n = 6 mice. (F, G) Mice were injected with 3-MC and analyzed after 3 d. (F) Representative flow cytometry plots of small intestinal eosinophils and epithelial cells from WT and Cyp1a1^Cre/+^Rosa26^YFP^ mice. (G) Frequency of YFP^+^ eosinophils and epithelial cells in the small intestine. Data are pooled from 2 independent experiments for n = 7 mice. (H–M) BMDEos were generated from *Ahrr*^EGFP/EGFP^ (H–J) or Cyp1a1^Cre/+^Rosa26^YFP^ mice (K–M) and WT controls. (I, J) BMDEos at day 13 of culture were treated with 5 nM FICZ or dimethyl sulfoxide control for 24 h, and the frequency of GFP^+^ cells was determined by flow cytometry. Data are pooled from 2 independent experiments for n = 6 biological replicates; mean ± SD is shown. (L, M) BMDEos were cultured to day 11 and then cultured for 3 d with 5 nM FICZ or dimethyl sulfoxide control. The frequency of YFP^+^ cells was determined by flow cytometry on day 14 of culture. Data are pooled from 3 independent experiments for n = 6 biological replicates. (J, M) Mean ± SD is shown. Eos = eosinophils; Mac = macrophages; MHC-II = major histocompatibility complex class II; SI = small intestine.

Single-cell suspensions were stained as described previously. For sorting multiple intestinal populations from the small intestine (Fig. 1), unselected cells were used to sort CD45^+^, EpCam^+^, and CD45^−^EpCam^−^ populations. Magnetic bead selection with anti-CD11b and anti-CD11c (Miltenyi Biotec) was performed according to protocol over LS columns to enrich myeloid cells (CD11b^+^ and/or CD11c^+^). These were sorted into macrophages, dendritic cells, and eosinophils (Fig. 1). For eosinophil sorting from the small intestine for RNA sequencing, cells were preselected with anti-CD11b microbeads and were additionally stained with DAPI (Sigma-Aldrich) to exclude any cells that died between staining and sorting. Cell sorting was conducted on Aria III or Fusion instruments (BD Biosciences) through a 100 µm nozzle using high-purity settings as previously described.^7^

### 2.8 RNA isolation

Cell pellets were resuspended in TRIzol (Invitrogen; #15596026) and RNA was isolated according to the manufacturer's protocol. For BMDEo culture time points and stimulated BMDEos, RNA was isolated from approximately 200,000 cells/sample. For RNA sequencing, RNA was isolated with TRI Reagent and the RiboPure RNA Purification Kit (Thermo Fisher Scientific; #AM1924) immediately after sorting. RNA quality and quantity was determined using the Agilent 2100 Bioanalyzer with the Agilent RNA 6000 Pico Kit. Only samples with RNA integrity number ≥7 were used for RNA sequencing. RNA isolation from eosinophils is difficult, due to their high RNase content. Only half of sorted eosinophil samples fulfilled the criterion of RNA integrity number ≥7, which could possibly introduce a bias in the sequencing results.

### 2.9 Quantitative polymerase chain reaction

RNA was reverse transcribed using the High-Capacity cDNA Reverse Transcription Kit (Thermo Fisher Scientific). The complementary DNA served as a template for the amplification of genes of interest and housekeeping genes by real-time quantitative polymerase chain reaction (qPCR), using TaqMan Gene Expression Assays (Applied Biosystems), universal PCR Master Mix (Applied Biosystems) and the QuantStudio 7 System (Applied Biosystems). Messenger RNA (mRNA) expression was determined using the ΔC_T_ method, normalizing to *Hprt* gene expression, and are shown as 2^−ΔCt^ to *Hprt*.

### 2.10 RNA sequencing and data processing

RNA samples were converted into complementary DNA using the NuGEN Ovation RNA-Seq System v2. Illumina-compatible libraries were produced using the NuGEN Ovation Ultralow Library System V2. Sequencing was performed on the Illumina HiSeq 4000 with single-ended reads of at least 75 bp. FastQ files were aligned to the GRCm38 Ensembl Release 86 mouse genome using the nextflow package nf-core/rnaseq to generate gene counts. Differential gene discovery was done using the bioconductor package DESeq2.^37^ Comparisons between WT, *Ahr*^−/−^, and *Ahrr*^−/−^ eosinophils were adjusted for sex. We recorded as differentially expressed those genes with adjusted *P* values <0.05. We also relied on DESeq2 for principal component analysis of variance–stabilized expression values. All computations were performed in R version 4.0.3 (2020-10-10; R Foundation for Statistical Computing). The dataset is deposited at GEO under accession GSE173831. WT and *Ahr*^−/−^ samples from this dataset have previously been analyzed.^7^

### 2.11 Hierarchical clustering

Hierarchical clustering was conducted using Morpheus software (https://software.broadinstitute.org/Morpheus) by clustering genes with an average linkage method and one minus Pearson correlation metric. For display, gene expression values were transformed using a relative color scheme showing row minimum to row maximum.

### 2.12 Gene Ontology analysis

Differentially expressed genes (adjusted *P* value <0.05) from different comparisons were analyzed using DAVID v6.8^38^ to determine Kyoto Encyclopedia of Genes and Genomes and Gene Ontology pathways and UniProt keywords. Selected pathways are shown.

### 2.13 Statistical analysis and graphing

Details of the statistical tests applied and the number of replicates are described in the corresponding figure legends. All data points and n values reflect biological replicates (mice). Statistical analysis and visualization was performed using GraphPad Prism 9 software (GraphPad Software).

## 3. Results

### 3.1 AHRR is highly expressed in intestinal eosinophils

We have previously shown that the transcription factor AHR is expressed and active in intestinal eosinophils and drives their transcriptional reprogramming.^7^ Given the regulation of AHR by negative feedback loops through AHRR and CYP1A1, we sought to determine if these proteins regulate AHR in intestinal eosinophils. We FACS-sorted eosinophils and other cell types from the small intestine of WT mice and assessed gene expression by qPCR (Fig. 1A, B). In line with our previous work,^7^ we found high *Ahr* expression in eosinophils. Relative to *Hprt*, expression levels of *Cyp1a1* were about 100-fold lower than *Ahrr* expression in eosinophils. In epithelial cells, which are known to regulate the availability of AHR ligands in the intestinal mucosa through enzymatic biotransformation,^13^ expression of *Cyp1a1* was higher than *Ahrr*. These data suggest that eosinophils express *Ahrr* over *Cyp1a1* among the canonical AHR target genes. Using a reporter mouse (*Ahrr*^EGFP/EGFP^), we found that while AHRR is not expressed in splenic eosinophils, about 70% of intestinal eosinophils were EGFP positive (Fig. 1A, B). In line with previous work on this strain,^31^ we found that a large proportion of intestinal macrophages were also AHRR-EGFP positive (Fig. 1C). In a reporter strain for CYP1A1 (*Cyp1a1*^Cre/+^*Rosa26*^YFP^ mice), we could not detect YFP^+^ eosinophils in the small intestine (not shown). Even after administration of the exogenous AHR ligand 3-MC, <1% of eosinophils were YFP^+^ (Fig. 1F, G). In contrast, small intestinal epithelial cells from the same mice readily expressed YFP. We also observed preferential expression of AHRR over CYP1A1 in BMDEos in vitro. In BMDEo cultures from *Ahrr*^EGFP/EGFP^ mice, about 20% of eosinophils were EGFP^+^ at day 14 of culture, and this could be increased to 50% when cells were cultured with the prototypical AHR ligand FICZ (Fig. 1H–J). It is important to note that cell culture medium contains a low level of AHR ligands,^39^ which may explain the presence of EGFP^+^ eosinophils even in the absence of FICZ stimulation. In contrast, in BMDEo cultures of *Cyp1a1*^Cre/+^*Rosa26*^YFP^ mice only about 2% of eosinophils were YFP^+^, and FICZ treatment increased this to about 7% (Fig. 1K–M). YFP expression in CYP1A1 reporter mice relies on CRE-mediated recombination of the Rosa26 locus, while the AHRR reporter is a direct knock-in of EGFP into the *Ahrr* locus. Therefore, a direct comparison of these two reporter lines is not possible, but the results still indicate that eosinophils preferentially induce AHRR over CYP1A1 in vitro and in vivo. This is contrary to intestinal epithelial cells, which induce CYP1A1 over AHRR.^13,31^

### 3.2 AHRR does not repress AHR-dependent genes in intestinal eosinophils

**Fig. 2.**
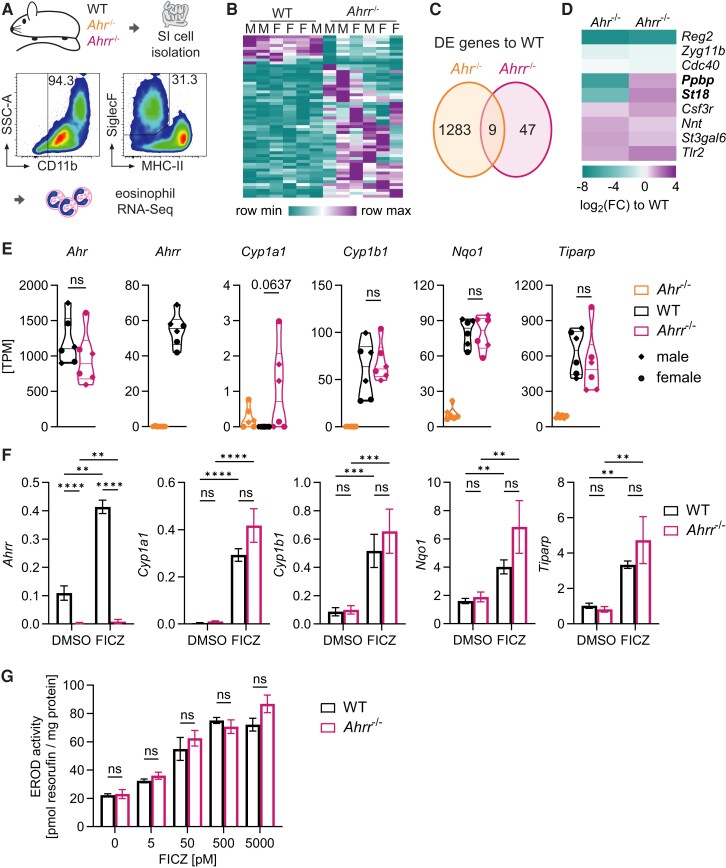
AHRR does not repress AHR-dependent genes in intestinal eosinophils. (A) Gating for small intestinal (SI) eosinophils following enrichment with CD11b microbeads. Cells are pregated on single, live, CD45^+^ cells. (B–E) RNA sequencing (RNA-Seq) of WT, *Ahr*^−/−^, and *Ahrr*^−/−^ eosinophils. n = 6 mice (3 males, 3 females) were analyzed per genotype. (B) Heatmap of 56 genes with differential expression (|log_2_(FC)|≥1 and adjusted *P* value <0.05) between eosinophils from WT and *Ahrr*^−/−^ mice. (C) Number of differentially expressed (DE) genes in *Ahr*^−/−^ vs. WT and *Ahrr*^−/−^ vs. WT eosinophils. Only 9 genes were found in both comparisons. (D) Heatmap of 9 genes with differential expression in both *Ahr*^−/−^ and *Ahrr*^−/−^ eosinophils. (E) Gene expression of canonical pathway genes in eosinophils from *Ahr*^−/−^, WT, and *Ahrr*^−/−^ mice. Data were analyzed by *t* test comparing *Ahrr*^−/−^ with WT eosinophils. (F, G) BMDEos were generated from WT and *Ahrr*^−/−^ mice. (F) Gene expression in BMDEos was determined by qPCR and normalized to *Hprt*. Data are from n = 4 to 5 biological replicates (mean ± SEM) and were analyzed by 2-way analysis of variance with Sidak correction. **P < 0.01; ***P < 0.001; ****P < 0.0001. (G) EROD activity in BMDEos from WT and *Ahrr*^−/−^ mice following treatment with FICZ or dimethyl sulfoxide (DMSO) control. Data were pooled from n = 3 independent experiments (mean ± SEM) and analyzed by *t* test with Holm-Sidak correction for multiple comparisons. F = female; M = male; MHC-II = major histocompatibility complex class II; ns = not significant; SSC-A = side scatter area; TPM = transcripts per million.

AHRR is thought to counteract the transcriptional activity of AHR, which led us to hypothesize that AHRR may regulate eosinophil gene expression in the intestine. We conducted RNA sequencing on FACS-sorted small intestinal eosinophils from WT, *Ahr*^−/−^, and *Ahrr*^−/−^ mice (Fig. 2A). We have previously reported in detail how AHR affects intestinal eosinophil gene expression and tissue adaptation,^7^ but what role AHRR plays in intestinal eosinophils is not known. We identified relatively few differentially expressed genes in *Ahrr*^−/−^ compared with WT eosinophils (n = 56) (Fig. 2B). Most differentially expressed genes were upregulated as compared with WT eosinophils, which is consistent with the role of AHRR as a transcriptional repressor. Based on the prevailing view in the literature, we expected to find that AHRR would repress AHR-dependent genes. However, only 9 genes were differentially expressed in both *Ahr*^−/−^ vs. WT and *Ahrr*^−/−^ vs. WT eosinophils (Fig. 2C). Among these, only 2 genes (*Ppbp* and *St18*) were downregulated in *Ahr*^−/−^ and upregulated in *Ahrr*^−/−^ eosinophils, as would be expected of genes that are dependent on AHR and repressed by AHRR (Fig. 2D). Several genes were upregulated in both *Ahr*^−/−^ and *Ahrr*^−/−^ eosinophils (*Csf3r*, *Tlr2*, *Nnt*, *St3Gal6*). We next looked in more detail at key AHR target genes. In the RNA sequencing dataset, no significant differences in the expression of *Ahr* or canonical AHR target genes could be found between WT and *Ahrr*^−/−^ eosinophils (Fig. 2E). *Cyp1a1* expression was undetectable in WT mice and detected at very low levels in 4 of 6 *Ahr*^−/−^ and 4 of 6 *Ahrr*^−/−^ mice, suggesting this was only a background level of expression. Next, we determined whether AHRR suppressed expression of canonical AHR target genes following stimulation with the AHR ligand FICZ. BMDEos were generated from WT and AHRR-deficient mice and gene expression analyzed by qPCR following FICZ stimulation (Fig. 2F). FICZ stimulation strongly induced AHR target gene expression in both WT and AHRR-deficient mice. As expected, *Ahrr* was largely undetectable in AHRR-deficient BMDEos. Other AHR-induced genes *Cyp1a1*, *Cyp1b1*, *Nqo1*, and *Tiparp* were induced at similar levels in WT and AHRR-deficient eosinophils. We also determined the enzymatic activity of CYP1A1 and CYP1B1, which biotransform AHR ligands as a negative feedback loop of the AHR signaling pathway. BMDEos lacking AHRR showed no increase in enzymatic activity compared with WT BMDEos across a range of FICZ concentrations (Fig. 2G). These data indicate that although AHRR is a transcriptional repressor in intestinal eosinophils, it does not seem to directly oppose the AHR-dependent gene expression program.

### 3.3 AHRR limits expression of antimicrobial genes in intestinal eosinophils

**Fig. 3.**
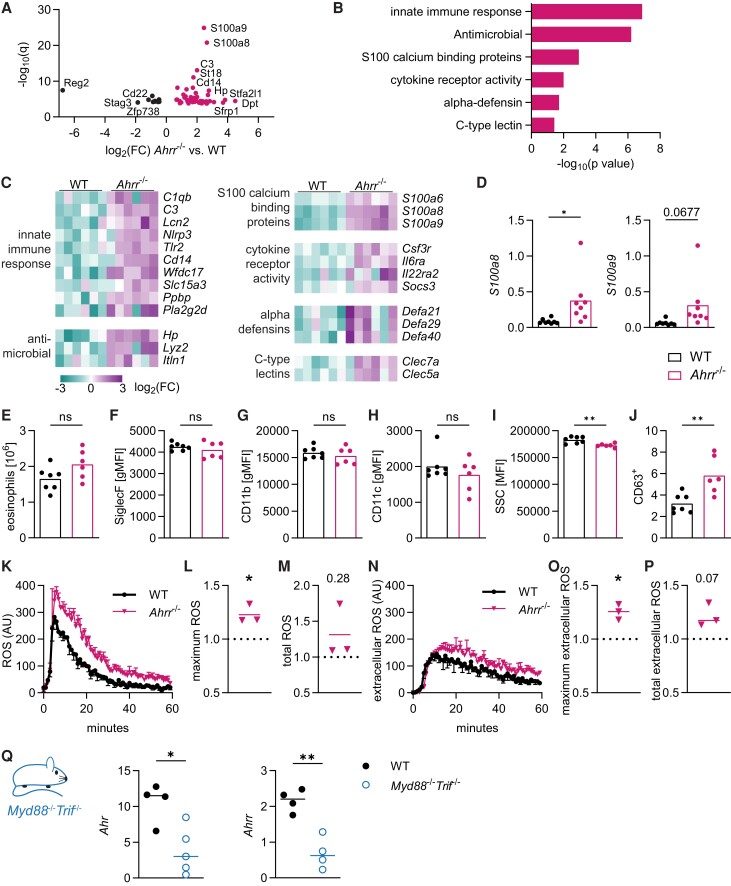
AHRR limits expression of antimicrobial genes in intestinal eosinophils. (A) Volcano plot of differentially expressed genes in *Ahrr*^−/−^ vs. WT intestinal eosinophils. (B) Gene Ontology analysis of differentially expressed genes was conducted using DAVID. (C) Heatmap of example genes within each enriched pathway. (D) Gene expression was determined by qPCR in sorted small intestinal eosinophils from WT and *Ahrr*^−/−^ mice and normalized to *Hprt*. Data are from n = 8 mice per genotype and were analyzed by *t* test. *P < 0.05. (E–J) Small intestinal eosinophils from WT and *Ahrr*^−/−^ mice were quantified and analyzed by flow cytometry. Data are pooled from 2 independent experiments for n = 6 to 7 mice per genotype. (K–P) Production of ROS in response to PMA stimulation in BMDEos from WT and *Ahrr*^−/−^ BMDEos. (K–M) Combined extracellular and intracellular ROS was measured with luminol. (N–P) Extracellular ROS was measured with isoluminol. (K, N) Representative plots of ROS production. (L, M, O, P) Maximal and total ROS production was determined in n = 3 independent experiments and normalized to WT BMDEos. Data were analyzed by 1-sample *t* test. *P < 0.05. (Q) Gene expression in sorted intestinal eosinophils was determined by qPCR and normalized to *Hprt*. Data are from 1 experiment with n = 4 mice per genotype and were analyzed by *t* test. *P < 0.05; **P < 0.01. AU = arbitrary units; gMFI = geometric mean fluorescent intensity; MFI = mean fluorescent intensity; SSC = side scatter.

Because we could not find evidence that AHRR generally limits the expression of AHR-dependent genes, we next sought to identify which pathways were regulated by AHRR. In Gene Ontology analysis, eosinophils from mice lacking AHRR showed particularly high expression of genes associated with the innate immune response, such as complement, inflammasome components, and pattern recognition receptors (Fig. 3A–C). AHRR deficiency also increased the expression of antimicrobial genes, the S100 calcium binding proteins S100a8 and S100a9, which we confirmed by qPCR (Fig. 3D), and alpha defensins. Eosinophils from *Ahrr*^−/−^ mice showed increased expression of genes in cytokine receptor signaling and increased expression of C-type lectins. No functional pathway enrichment could be identified for the 10 downregulated genes in *Ahrr*^−/−^ eosinophils. We characterized eosinophils by flow cytometry to determine if they showed an activated phenotype. There was no difference in eosinophils numbers or expression of SiglecF, CD11b, or CD11c on small intestinal eosinophils between WT and *Ahrr*^−/−^ mice (Fig. 3E–H). However, *Ahrr*^−/−^ eosinophils had reduced side scatter and increased CD63 surface expression (Fig. 3I, J), signs of increased degranulation in the intestine. On the functional level, we found that AHRR-deficient BMDEos had increased production of reactive oxygen species (ROS), which are part of the antimicrobial defense (Fig. 3K–P). Increased ROS production was only seen after PMA stimulation but not in unstimulated eosinophils. These findings imply that AHRR might function to limit the innate antimicrobial immune response. We hypothesized that if AHRR regulates the response to bacteria, then microbial signals might play a role to induce *Ahr* and *Ahrr*. *Myd88*^−/−^*Trif*^−/−^ mice lack the ability to sense many pathogen-associated molecular patterns through toll like receptors.^34,40,41^ We found that both *Ahr* and *Ahrr* were decreased in intestinal eosinophils of *Myd88*^−/−^*Trif*^−/−^ mice (Fig. 3Q). This suggests that microbial signals can induce *Ahr* and *Ahrr* gene expression. It has been previously shown that lipopolysaccharide (LPS) can induce *Ahr* and *Ahrr* expression and AHR plays an important role in protecting mice from LPS-induced shock.^31,42,43^ It is possible that AHR-mediated induction of AHRR and the subsequent suppression of innate inflammatory and antimicrobial response genes are key to prevent overactivation of the host organism. Together, this supports the idea that AHRR plays a role in limiting the expression of innate immune response and antimicrobial genes, possibly to maintain an anti-inflammatory phenotype in eosinophils when exposed to microbial signals in the intestinal environment.

## 4. Discussion

In the intestine, AHRR is important to maintain intraepithelial lymphocytes, limit production of IL-1b from T helper 17 cells, and dampen intestinal inflammation.^31,32^ AHRR is expressed at particularly high levels in intestinal myeloid cells, such as macrophages and dendritic cells,^31^ and as we showed previously, also in eosinophils.^7^ Here, we could demonstrate that AHRR in intestinal eosinophils restricts the expression of innate immune response and antimicrobial genes, such as alpha defensins, S100 proteins, C-type lectins, and pattern recognition receptors. Intestinal immune cells are constantly exposed to microbial signals from the microbiota. AHRR may be key to restrain the antimicrobial and proinflammatory response in eosinophils when exposed to microbial signals in the intestinal environment.

We found that AHRR expression in eosinophils is tissue dependent. In AHRR reporter mice, about 70% of intestinal eosinophils but no splenic eosinophils were GFP^+^. This confirmed previous comparison of *Ahrr* mRNA expression across different tissue eosinophils^7^ and is in line with other myeloid cells, which express high levels of AHRR in barrier organs such as skin or intestine but not in the spleen.^31^ In addition, we could show that eosinophils, like other intestinal myeloid cells, preferentially express *Ahrr* over *Cyp1a1*. Expression levels were about 100-fold higher for *Ahrr* than *Cyp1a1*, which was barely detectable in our RNA sequencing data of small intestinal eosinophils. Intestinal epithelial cells and CD45^−^EpCam^−^ stromal cells (including fibroblasts and endothelial cells) assayed in parallel had relatively higher expression of *Cyp1a1* over *Ahrr*. This is consistent with the previous recognition that *Ahrr* is predominantly expressed in immune cells of the skin and intestine of mice while *Cyp1a1* is expressed in epithelial cells of these tissues.^13,31,44^ There appears to be a clear dichotomy between induction of AHRR vs. CYP1A1 in response to AHR activation in different cell types.

Unexpectedly, we did not find evidence for a role of AHRR in limiting the expression of AHR-dependent genes. Only 2 (*St18*, *Ppbp*) of the over 1000 AHR-induced genes^7^ were repressed by AHRR. In particular, we could not find evidence for AHRR-mediated repression of *Cyp1a1*, *Cyp1b1*, *Nqo1*, or *Tiparp* mRNA in small intestinal eosinophils or CYP1A1/B1 enzymatic activity in cultured eosinophils. In many other cell types, AHRR has been shown to suppress *Cyp1a1*, *Cyp1b1*, and *Tiparp* mRNA expression. This has been reported at the whole tissue level,^45^ in mouse embryonic fibroblasts,^46^ and most recently in intraepithelial lymphocytes in the intestine.^32^ In intraepithelial lymphocytes, AHRR deficiency increased not only mRNA expression, but also enzymatic activity of CYP1A1, and as a result increased ROS production as a by-product.^32^ In other cell types, however, such as human skin fibroblasts and mouse embryonic fibroblasts, AHRR does not correlate with CYP1A1 enzymatic activity.^30^ Our data suggest that AHRR functions beyond repression of AHR canonical activity are likely and might influence other signaling pathways. AHRR has been shown to interfere with noncanonical AHR-mediated signaling by interacting with RelB and other transcription factors, and thereby inhibiting inflammatory responses.^29,47,48^ It is possible that AHRR can take on cell type–specific functions that are more complex than previously thought.

We found increased ROS production in AHRR-deficient eosinophils in vitro. However, unlike the report on intraepithelial lymphocytes,^32^ increased ROS production in eosinophils did not stem from increased CYP1A1 enzymatic activity, which was unchanged by the absence of AHRR. Moreover, unlike intraepithelial lymphocytes, eosinophil numbers were not reduced in *Ahrr*^−/−^ mice. It is possible that eosinophils are less sensitive to oxidative stress than intraepithelial lymphocytes. They are capable of generating large amounts of ROS through eosinophil peroxidase and other granule proteins and may therefore have strong ROS scavenging mechanisms in place to protect cellular viability.^49,50^ It remains to be seen if the regulation of ROS production by AHRR is specific to eosinophils or a general function in myeloid cells.

While many intestinal cell types express high levels of AHR, it is unknown which factors drive AHR expression. Here, we could show that *Myd88*^−/−^*Trif*^−/−^ mice had reduced expression of *Ahr* and *Ahrr* in small intestinal eosinophils. These mice lack the ability to sense multiple pathogen associated patterns, such as those signaling through TLRs and IL-1 receptor families, suggesting that microbial signals play a role in inducing *Ahr*/*Ahrr* expression in intestinal eosinophils. The TLR4 ligand LPS has previously been shown to induce *Ahr* expression in peritoneal macrophages.^42,43^ As an AHR target gene, *Ahrr* expression is also highly dependent on AHR expression as well as on AHR ligands and can be induced in intestinal immune cells through dietary AHR ligands.^16^ Our study did not investigate how availability of dietary or xenobiotic AHR ligands alter *Ahrr* expression or downstream function. It is possible that the presence of microbial signals and increased microbial-derived AHR ligands^51^ might also induce a strong AHRR and antimicrobial response. The exact mechanisms that control AHR and AHRR expression in intestinal eosinophils and other immune cells remain to be discovered.

In a systemic LPS-induced shock model, AHR-deficient mice are more susceptible while AHRR-deficient mice showed increased survival.^31,42,43^ This contrasts with experimental dextran sulfate sodium–induced colitis, in which both AHR- and AHRR-deficient mice exhibit increased disease severity, indicating that the local tissue environment affects the role of AHRR in limiting or promoting inflammatory reactions. Increasing evidence shows that AHR and AHRR also play important context-dependent roles in cancer, potentially functioning as tumor suppressors or by dampening the antitumor immune response.^12,52–54^ Our study showed that AHRR may be key to maintain an anti-inflammatory phenotype and limit the antimicrobial response in eosinophils when exposed to microbial signals in the intestinal environment. It remains to be examined if eosinophil-specific AHRR deletion leads to changes in susceptibility of mice to intestinal bacterial infection or the microbiota composition. Lack of AHR in intestinal eosinophils affects the local immune system and increases susceptibility to helminth infection, but whether it alters the microbiome or immune response to bacterial infection is not known.^7,8^ Because AHR is known to promote an anti-inflammatory phenotype in myeloid cells, it can be speculated that AHRR and its suppression of innate immune response genes is part of the broader AHR-mediated regulation of immune cells in barrier organs.
